# Depression and Incident Dementia. An 8-Year Population-Based Prospective Study

**DOI:** 10.1371/journal.pone.0059246

**Published:** 2013-03-19

**Authors:** Melanie Luppa, Tobias Luck, Franziska Ritschel, Matthias C. Angermeyer, Arno Villringer, Steffi G. Riedel-Heller

**Affiliations:** 1 Institute of Social Medicine, Occupational Health and Public Health, University of Leipzig, Leipzig, Germany; 2 Douglas Mental Health University Institute, McGill University, Montreal, Canada; 3 Center for Public Mental Health, Goesing a. W., Austria; 4 Department of Public Health, University of Cagliary, Cagliary, Italy; 5 Max Planck Institute for Human Cognitive and Brain Sciences and Day Clinic of Cognitive Neurology, University of Leipzig, Leipzig, Germany; 6 LIFE – Leipzig Research Center for Civilization Diseases, University of Leipzig, Leipzig, Germany; University of Pennsylvania, United States of America

## Abstract

**Aims:**

The aim of the study was to investigate the impact of depression (categorical diagnosis; major depression, MD) and depressive symptoms (dimensional diagnosis and symptom patterns) on incident dementia in the German general population.

**Methods:**

Within the Leipzig Longitudinal Study of the Aged (LEILA 75+), a representative sample of 1,265 individuals aged 75 years and older were interviewed every 1.5 years over 8 years (mean observation time 4.3 years; mean number of visits 4.2). Cox proportional hazards and binary logistic regressions were used to estimate the effect of baseline depression and depressive symptoms on incident dementia.

**Results:**

The incidence of dementia was 48 per 1,000 person-years (95% confidence interval (CI) 45–51). Depressive symptoms (Hazard ratio HR 1.03, 95% CI 1.01–1.05), and in particular mood-related symptoms (HR 1.08, 95% CI 1.03–1.14), showed a significant impact on the incidence of dementia only in univariate analysis, but not after adjustment for cognitive and functional impairment. MD showed only a significant impact on incidence of dementia in Cox proportional hazards regression, but not in binary logistic regression models.

**Discussion:**

The present study using different diagnostic measures of depression on future dementia found no clear significant associations of depression and incident dementia. Further in-depth investigation would help to understand the nature of depression in the context of incident dementia.

## Introduction

The relationship of depression and dementia is complex, and several assumptions about the role of depression in relationship to the development of dementia are discussed: depression as an early prodromal clinical manifestation of dementia, as an etiologic risk factor, or as an early reaction to cognitive decline [1]–[3]. Currently available evidence suggests that depression in particular may constitute an etiologic risk factor of dementia, even though no consensus was found on this point so far [4].

A series of prospective studies investigated the impact of depression on the incidence of dementia [5]–[13]. Studies, however, differed greatly in their methodical approaches and showed inconsistent results. To some extent this may be due to varying diagnostic measures for depression. Studies used categorical approaches identifying syndrome clusters on the basis of ICD-10 or DSM-IV diagnostic criteria to diagnose major depression (MD) or dimensional measures to quantify the severity of depressive symptoms and to diagnose different subtypes and subsyndromal stages. Moreover, several studies investigated specific patterns of depressive symptoms (i.e. mood or motivational) and its impact on incident dementia [6]; [11]; [12]; [14], since a predominance of either mood- or motivation-related symptoms may highlight the nature of depressive signs in relationship to development of dementia.

The present study aims to evaluate the impact of different diagnostic measures of depression on future dementia in the very same sample; thereby other methodical differences which may have affected the outcomes in previous studies will be equalized. We aim to investigate particularly the impact of (1) depression (categorical diagnosis of MD) and (2) depressive symptoms (dimensional measure and symptom pattern) on future dementia within an 8-year German prospective population-based representative study.

## Methods

### 1. Ethics Statement

The study was approved by the Ethics committee of Leipzig University (Ethik-Kommission an der Medizinischen Fakultät der Universität Leipzig). Since it was an observational study with face-to-face assessments in the home environment, written informed consent of the participants was obtained before assessment. Their capacity to consent was judged by special trained and experienced physicians and psychologists before starting the interview, thus clinician-based. In cases where capacity to consent was doubted, informed consent was obtained by next of kin, care takers as well as guardians on the behalf of participants.

### 2. Sample

The Leipzig Longitudinal Study of the Aged (LEILA 75+) is a populations-based prospective study of a large cohort of older adults in Leipzig, Germany. At baseline in 1997/98, a total of 1,692 subjects aged 75 years and over were enrolled in the sample (age range = 75–99 years, mean age = 81.5). Of these, 1,500 subjects were identified by systematic random sampling from an age-ordered list provided by the local registry office. In addition, institutionalised subjects were included in the study by proportion (n = 192). The study design of the LEILA 75+ has been described in detail elsewhere [15]–[17].

### 3. Study Setting and Assessment Procedures

Upon enrolment, information was given by mail; further contact was made by telephone or by home visit to ask for approval. During the baseline visit, face-to-face interviews were conducted by trained physicians and psychologists at the participants’ homes. With regard to cognitively impaired participants, additional structured third-party interviews were conducted with proxies. Follow-up interviews were carried out for all eligible participants every 1.5 years during the study period of 8 years. The ethics committees of the University of Leipzig approved the study. Written informed consent was obtained from all participants.

### 4. Instruments

*Socio-demographic variables* were recorded with a standardized questionnaire including age, gender, marital status, and educational level (based on the revised version of the international CASMIN educational classification [18]).

*Functional and cognitive impairment:* The ability to perform basic and complex (instrumental) activities of daily living (ADL/IADL) was assessed with the 26-item ADL/IADL scale [19], which was developed according to an internationally used IADL list [20]. Cognitive status was assessed with the 30 items of the Mini-Mental State Examination (MMSE) [21].

### 5. Diagnosis of Depression and Depressive Symptoms

***Depressive symptoms*** were dimensionally administered with the German version of the 20-item Center of Epidemiologic Studies Depression Scale (CES-D) [22]; [23]. A dimensional diagnosis of depression was considered as being present, if the participants scored 23 or more points on the CES-D [23]. The psychometric properties of the German version of the CES-D were evaluated among young adults [23] as well as in the elderly population [24]. Both studies reported a high internal consistency and construct validity of the CES-D. If a participant did not respond to fewer than 6 items of the CES-D, missing values were replaced by the mean score of the responded items; if 6 or more items were missing, the CES-D could not be evaluated [22]. Since advanced cognitive impairment significantly interferes with the reliability and validity of the CES-D, the CES-D was not administered to participants with a MMSE score of less than 19 points [25].

Previous studies reported on a differential impact of ***mood-related*** and ***motivation-related symptoms of depression*** on incident dementia. We therefore also assessed the effect of these two symptom patterns on dementia (according to recommendations of [6]); mood-related symptoms included the CES-D items blues (3.), depressed (6.), happy (12.), cry (17.) and sad (18.), and motivation-related symptoms the items effort (7.), talk (13.) and get going (20.).

Categorical diagnosis of ***Major Depression*** was assessed by means of the Structured Clinical Interview (SCID) for DSM-III-R [26].

### 6. Definition of Dementia

Dementia was assessed by means of the SIDAM (Structured Interview for Diagnosis of Dementia of Alzheimer type, Multi-infarct Dementia and Dementia of other Aetiology according to DSM-III-R, DSM-IV and ICD-10 [27]) according to DSM-IV criteria [28]. The SIDAM consists of (a) a cognitive test battery and (b) a section for clinical judgement and third-party information on psychosocial impairment, including a scale for the assessment of activities of daily living with 14 items (SIDAM-ADL scale). The cognitive test battery consists of 55 items, including the 30 items of the MMSE [21]. The criteria of a full work up of the SIDAM were an MMSE score lower than 24, and at least two impairment in the SIDAM-ADL scale. If it was not possible to administer the SIDAM at a follow-up assessment (e.g. because of death or severe weakness), a comprehensive structured proxy interview was offered including the Clinical Dementia Rating Scale (CDR [29]). For each subject, consensus conferences of physicians and psychologists were held.

### 7. Statistical Analyses

The statistical analyses were performed with SPSS for Windows, version 20.0. Group differences were analyzed with t-test and χ^2^-test as appropriate. Incidence of dementia was calculated as the number of cases diagnosed as demented during the follow-up waves divided by the person-years at risk. Incident dementia could only be diagnosed at the defined times of follow-up assessments. On average, the exact time of first possible diagnosis could be assumed at the midway point between the follow-up visit, when dementia was diagnosed, and the previous visit. Person-years at risk were calculated accordingly. For those subjects who did not develop a dementia at the follow-up waves, person-years at risk were calculated as the time between the baseline visit and the last follow-up interview the participant had attended. We estimated time to incident dementia using Kaplan-Meier survival analysis. Log Rank test was used to compare survival distributions of time to incident dementia of subgroups.

We used univariate and multivariate Cox proportional hazards regression models to examine the impact of depression and depressive symptoms on time to incident dementia: firstly, we estimated the impact of depressive symptoms in terms of a dimensional diagnosis (CES-D total score); secondly, the impact of especially mood- and motivation-related symptoms of the CES-D (mood- and motivation-related CES-D score); and thirdly, the impact of MD as categorical diagnosis (SCID). All multivariate models were adjusted for age (per year), gender, educational level (low, middle, high), marital status (married, single, divorced, widowed), functional impairment (ADL/IADL performance; 0– no problems, 1– with difficulties, 2– impossible), and cognitive status (MMSE score) at baseline. For each variable, Hazard Ratios (HR) and 95% Confidence Intervals (CI) were calculated. Additionally, post-hoc analytic, binary logistic regressions were conducted to prove the effects of depression and depressive symptoms on risk of incident dementia leaving the time until dementia diagnosis out of consideration. For each variable, Odds Ratios (OR) and 95% Confidence Intervals (CI) were calculated. Risk factor analyses were done hypothesis driven. The significance level was set at 0.05 (two-tailed) for all analyses.

## Results

### 1. Baseline Assessment

**Figure 1:**
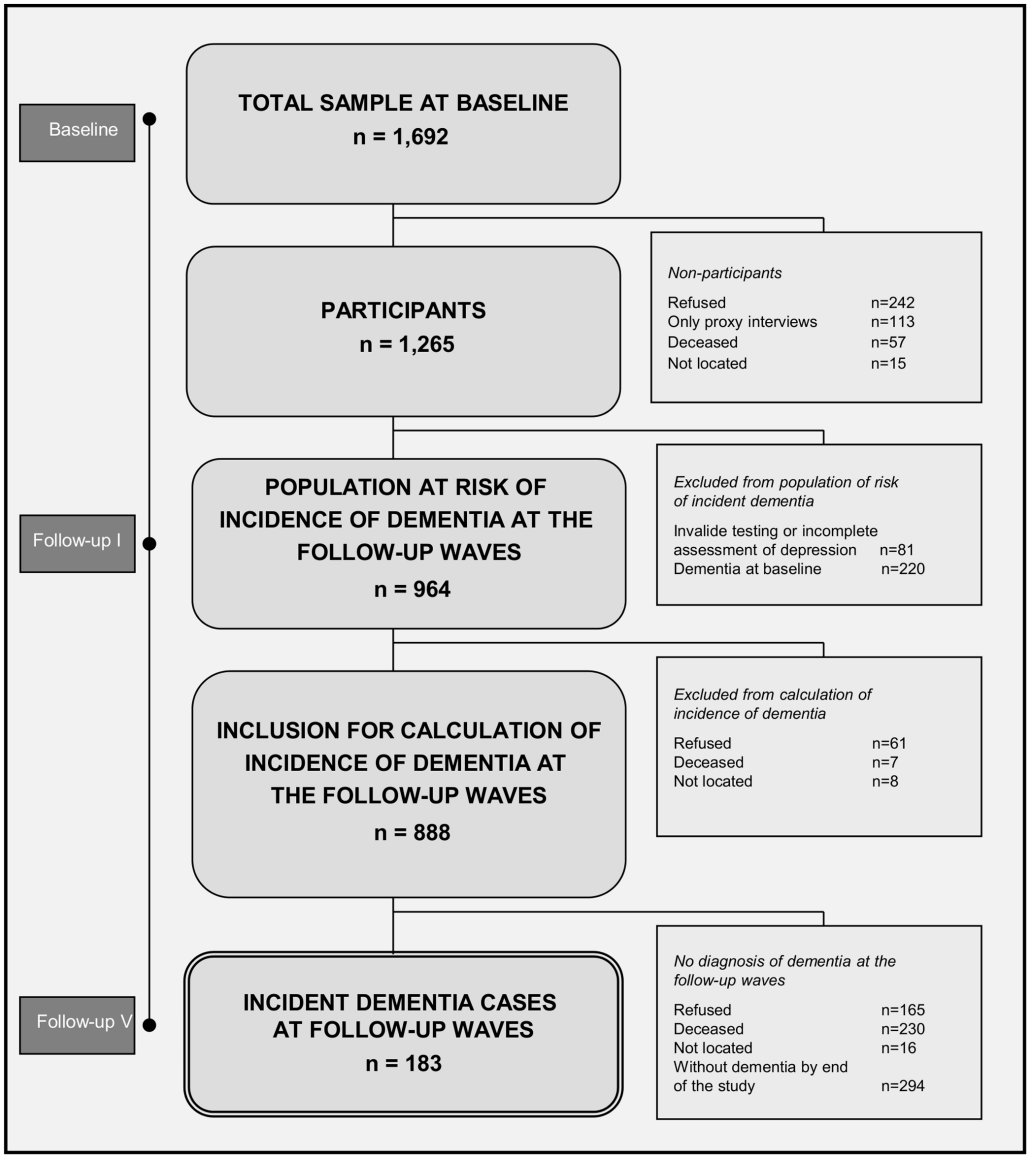
Sampling Frame.

Of the total sample of 1,692 individuals eligible to participate, 242 (14.2%) subjects refused participation, 57 (3.4%) died before they could be enrolled, and 15 (0.9%) could not be located; information on 113 (6.7%) individuals shielded by their relatives was obtained solely by proxy interviews (see fig. 1). The response rate at baseline was 74.8%, resulting in a sample of 1,265 subjects. These 1,265 subjects did not differ from the remainder of the sample in terms of age (U = 263,493, p = 0.451), gender (χ^2^ = 0.391, p = 0.532) or marital status (χ^2^ = 5.027, p = 0.170).

Of the 1,265 participants at baseline, 220 were already classified as having dementia, and 81 had invalid or incomplete assessment of depression (less than 19 in the MMSE; 6 or more missing items in the CES-D). Of the remaining 964 participants, information on at least one follow-up up to five follow-up waves was available for 888 (92.1%). The average (SD) time of follow-up was 4.3 (2.4) years; the average (SD) number of visits was 4.2 (1.6). The analysis of the impact of depression and depressive symptoms on incident dementia is based on these 888 participants.

### 2. Incidence of Dementia

During a follow-up of 3,830 person-years at risk, 183 participants developed dementia (incidence rate: 48 per 1,000 person-years, 95% CI 45–51), amounting to an annual risk of 4.8%. Table 1 shows the baseline characteristics of the participants with and without incident dementia at the follow-up waves. Participants with incident dementia were significantly older, more often females, more often widowed, and showed higher functional and cognitive impairment at baseline than participants without.

**Table 1:** The risk of incident dementia during follow-up in 888 participants aged 75 years and older without dementia at baseline.

Characteristic	Subcategory,	No incident dementia n = 705	Incident dementia n = 183	*p* value	Univariate hazard ratio (95% CI)	*p*-value	Full models: hazard ratio (95% CI), fully adjusted for all variables					
							Model I:	*p* value	Model II:	*p* value	Model III:	*p* value
Age,	mean (s.d.)	80.6 (4.5)	83.8 (4.7)	**<0.001**	1.16 (1.12–1.19)	**<0.001**	1.10 (1.06–1.13)	**<0.001**	1.09 (1.06–1.13)	**<0.001**	1.10 (1.06–1.13)	**<0.001**
Gender, n (%)	Female	499 (70.8)	153 (83.6)	**<0.001**	1.74 (1.18–2.58)	**0.005**	1.30 (0.82–2.04)	0.263	1.28 (0.82–2.02)	0.281	1.27 (0.81–2.00)	0.294
Educational level^1^, n (%)	Low	444 (63.2)	127 (69.4)	0.170	1	–	1	–	1	–	1	–
	Middle	165 (23.5)	40 (21.9)		0.84 (0.59–1.20)	0.348	1.31 (0.91–1.89)	0.151	1.31 (0.91–1.89)	0.153	1.32 (0.91–1.90)	0.142
	High	94 (13.4)	16 (8.7)		0.56 (0.34–0.95)	**0.030**	1.77 (1.00–3.15)	0.051	1.79 (1.01–3.17)	**0.048**	1.84 (1.04–3.27)	**0.038**
Marital status, n (%)	Married	221 (31.3)	29 (15.8)	**<0.001**	1	–	1	–	1		1	
	Single	65 (9.2)	13 (7.1)		1.39 (0.72–2.66)	0.330	0.76 (0.38–1.54)	0.448	0.76 (0.38–1.55)	0.453	0.78 (0.39–1.59)	0.496
	Divorced	60 (8.5)	15 (8.2)		1.75 (0.94–3.26)	0.080	1.13 (0.57–2.22)	0.732	1.11 (0.56–2.19)	0.760	1.11 (0.57–2.19)	0.754
	Widowed	359 (50.9)	126 (68.9)		2.46 (1.64–3.68)	**<0.001**	1.34 (0.85–2.12)	0.213	1.33 (0.84–2.11)	0.221	1.36 (0.86–2.16)	0.186
Performance in ADL/IADL	mean (s.d.)	0.19 (0.30)	0.40 (0.42)	**<0.001**	–	–	–	–	–	–	–	–
	per one point	–	–	–	4.84 (3.58–6.55)	**<0.001**	2.75 (1.90–3.98)	**<0.001**	2.71 (1.86–3.94)	**<0.001**	2.66 (1.84–3.85)	**<0.001**
Cognitive state, MMSE	mean (s.d.)	27.4 (1.9)	25.9 (2.1)	**<0.001**	-	-	-	-	-	-	-	**-**
	per one point	–	–	–	0.71 (0.67–0.76)	**<0.001**	0.76 (0.71–0.82)	**<0.001**	0.76 (0.71–0.82)	**<0.001**	0.76 (0.71–0.81)	**<0.001**
Depressive symptoms^2^ CES-D	mean (s.d.)	14.3 (7.6)	15.8 (7.0)	**0.015**	–	–	–	–	–	–	–	–
	per one point	–	–	–	1.03 (1.01–1.05)	**0.004**	1.00 (0.98–1.02)	0.629	–	–	–	–
Mood-relatedsymptoms	mean (s.d.)	3.4 (2.6)	4.1 (2.7)	**0.004**	–	–	–	–	–	–	–	–
	per one point	–	–	–	1.08 (1.03–1.14)	**0.002**	–	–	1.00 (0.94–1.06)	0.956	–	–
Motivation-related symptoms	mean (s.d.)	1.9 (1.7)	2.1 (1.8)	0.241	–	–	–	–	–	–	–	–
	per one point	–	–	–	1.06 (0.97–1.15)	0.212	–	–	1.00 (0.90–1.10)	0.951	–	–
Major depression (SCID, DSM-IV)^3^	n (%)	7 (1.0)	4 (2.2)	0.194	2.60 (0.97–7.02)	0.059	–	–	–	–	2.75 (1.01–7.50)	**0.048**

Abbreviations: ADL – Activities of daily living; CES-D – Center for Epidemiologic Studies Depression Scale; CI – Confidence interval; IADL – Instrumental activities of daily living; MMSE – Mini Mental State Examination; SCID – Structured Clinical Interview; s.d. – standard deviation.

Notes:

1 Based on the revised version of the international CASMIN educational classification;

2 128 participants (14.4%) with depressive symptoms at baseline (CES-D cut-off score 23);

3 11 participants (1.2%) with major depression at baseline.

### 3. Impact of Depression and Depressive Symptoms on Incident Dementia

Of the 128 participants (14.4%) with depressive symptoms (CES-D cut-off score 23, [23]), and of the 11 participants (1.2%) with MD at baseline, 23.4% (n = 30) and 36.4% (n = 4) developed dementia during the study course, compared to 20.1% and 20.4% of those without depressive symptoms or MD, respectively. The participants who developed dementia over the study course showed a significantly higher mean CES-D score (15.8 vs. 14.3, p = 0.015) and a higher mood-related symptom score (4.1 vs. 3.4, p = 0.004) than those without. Major depression was also more often in those with incident dementia than in those without; the difference, however, failed to be significant.

No significant differences related to age, gender and MMSE score between those with and without depressive symptoms or MD in relationship to the development of dementia over the study course were found, except for those participants with depressive symptoms without developing dementia. They were more often female and had a lower MMSE score at baseline than those without (see table 2).

**Table 2:** Characteristics of individuals with and without depressive symptoms and with and without major depression at baseline according to incident dementia diagnosis (n = 888).

Characteristic	*No incident dementia (n = 705)*						*Incident dementia ( = 183)*					
	With depressive symptoms (n = 98)	Without depressive symptoms (n = 607)	*p* value	With major depression (n = 7)	Without major depression (n = 698)	*p* value	With depressive symptoms (n = 30)	Without depressive symptoms (n = 153)	*p* value	With major depression (n = 4)	Without major depression (n = 179)	*p* value
Age, mean (s.d.)	81.4 (5.0)	80.5 (4.4)	0.069	81.5 (5.3)	80.6 (4.5)	0.592	83.9 (4.5)	83.8 (4.7)	0.982	82.0 (2.8)	83.9 (4.7)	0.271
Female gender, n (%)	88 (89.8)	411 (67.7)	**<0.001**	7 (100.0)	492 (70.5)	0.088	27 (90.0)	126 (82.4)	0.301	3 (75.0)	150 (83.8)	0.638
Cognitive state: MMSE, mean (s.d.)	27.0 (2.1)	27.5 (1.9)	**<0.05**	27.1 (1.3)	27.4 (1.9)	0.714	25.8 (2.1)	25.9 (2.1)	0.827	25.8 (0.5)	25.9 (2.1)	0.870

Results of the univariate and multivariate Cox proportional hazards regression models of the impact of depression and depressive symptoms on time to incident dementia are shown in table 1. In univariate analyses, a significant effect on time to incident dementia was found for total CES-D score and mood-related symptoms, but not for motivation-related symptoms and MD. In multivariate regression models adjusted for age, gender, education, marital status, functional and cognitive impairment, a significant effect was only found for MD. With MD at baseline, the risk of incident dementia almost increased threefold (HR = 2.75; 95% CI 1.01–7.50).

**Figure 2:**
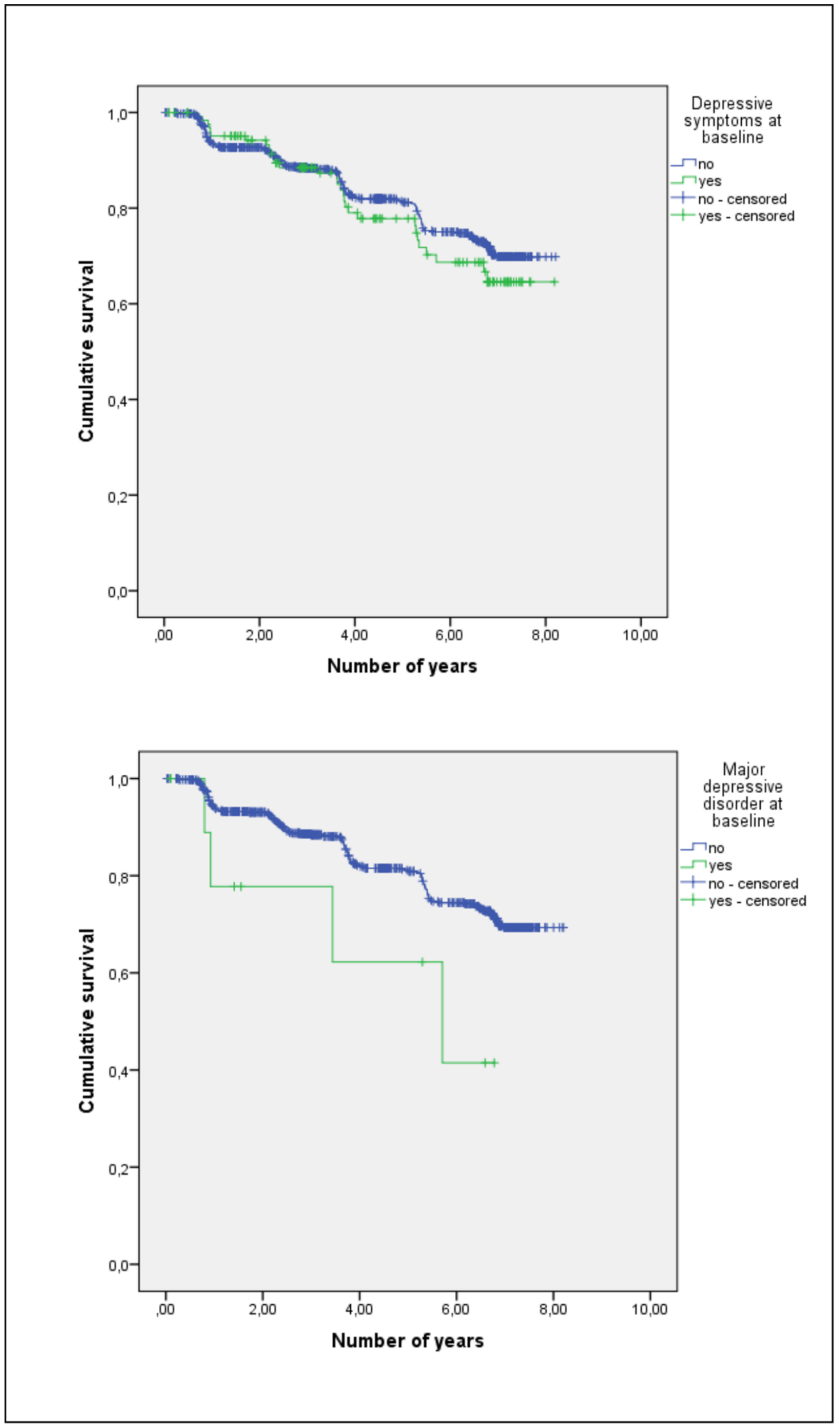
Kaplan-Meier survival curves of depressive symptoms and major depression.

Figure 2 presents the Kaplan-Meier survival curves for individuals with and without MD, and with and without dimensional diagnosis of depression (CES-D score ≥23) at baseline. Mean time to incident dementia was significantly shorter for participants with MD than for those without (4.7 years, 95% CI 3.1–6.3 vs. 6.8 years, 95% CI 6.7–7.0, Log rank: χ^2^ = 3.8 p = 0.049). By contrast, no significant difference was found between the mean time of participants with and without dimensional diagnosis (6.6 years, 95% CI 6.2–7.1 vs. 6.8 years, 95% CI 6.7–7.0, Log rank: χ^2^ = 0.7 p = 0.411).

### 4. Post-hoc Analyses

The effects of different diagnostic measures of depression on risk of incident dementia were analysed additionally by use of binary logistic regression models neglecting the time to development of dementia. In univariate binary logistic regression models similar results like in the Cox proportional hazard regression models for total CES-D score (OR 1.03, 95% CI 1.01–1.05, p = .016), mood- (OR 1.09, 95% CI 1.03–1.16, p = 0.005) and motivation-related symptoms (OR 1.06, 95% CI 0.97–1.16, p = 0.226), and MD (OR 2.23, 95% CI 0.65–7.70, p = 0.205) were found. The fully adjusted binary logistic regression models showed similar results for total CES-D (OR 0.99, 95% CI 0.97–1.02, p = 0.590), and mood- (OR 1.00, 95% CI 0.93–1.08, p = 0.990) and motivation-related symptoms (OR 1.01, 95% CI 0.90–1.14, p = 0.828). For MD the effect on risk of incident dementia disappeared if time to dementia diagnosis was not included in the regression model (OR 1.70, 95% CI 0.45–6.37, p = 0.431).

Furthermore, we analysed the additional impact of intermediate stages on the pathway from normal cognition to dementia by using the diagnosis of mild cognitive impairment (MCI) at baseline according to the international widely used criteria of [30] conducting separate regression analyses and Kaplan-Meier survival analyses for those with and without an MCI diagnosis. No differences related to the impact of depressive symptoms on time to incident dementia were found, however, a considerable difference of impact of MD between those with and without an MCI diagnosis at baseline (HR 6.15, 95% CI 1.88–20.13, p = 0.003 vs. HR 0.96, 95% CI 0.13–6.85, p = 0.965; time until dementia 1.72 vs. 5.30, Log rank: χ^2^ = 11.8 p = 0.001).

## Discussion

The aim of the study was to investigate the impact of depressive symptoms (dimensional diagnosis and symptom patterns) as well as depression (categorical diagnosis, major depression) on incident dementia in the German general population.

Participants who developed dementia over the study course of 8 years had a higher mean CES-D total score and in particular a higher mean CES-D score in mood-related symptoms at baseline. Both a higher CES-D total score and a higher score of mood-related symptoms were also found to have a significant impact on time to incident dementia in univariate regression models. By contrast, no significant univariate impact was found for motivation-related symptoms. These findings are in line with the findings of other studies [11]; [12]. Devanand et al. [11] investigated depressed mood as a risk factor of incident dementia using the depressed mood item of the Hamilton Rating Scale for Depression and found a twofold increased risk of incident dementia. Geerlings et al. [12] also reported a stronger effect of depressive symptoms on incident dementia when only affective symptoms were included in the analyses. Contrary to these findings, Berger et al. [6], showed a significant effect for motivational symptoms, including symptoms such as lack of interest, loss of energy, and concentration difficulties, on incident dementia. However, in this study also one of the mood-related symptoms (thoughts of death) showed a significant impact on incidence of dementia, and the mood factor failed to reach significance only slightly. With these not quite consistent findings, a definite answer to the question whether a specific symptom pattern in particular might increase the risk of incident dementia is still pending.

However, the univariate effects of depressive symptoms on development of dementia disappeared after adjustment for socio-demographic characteristics, cognitive and functional state. Likewise a part of studies in this research area did not found effects of depressive symptoms on development of dementia even after adjustment for covariates especially for cognitive state [6]; [10], while others did [5]; [7]–[9]; [11]; [12]; [14], but one study only in men [9], and another only in higher educated individuals [12]. The inconsistent findings of studies of the association between depression and dementia are discussed in relationship to the potential different underlying conditions for depressive mood including psychiatric disorders, neurodegenerative processes as well as severe medical comorbidity or bereavement [1]; [3]; [31]; [32], and further studies offering transparency related to study design and statistical analytic strategy, drawing on replications with larger samples to ascertain results from smaller studies and ensuring availability of all relevant data (as recently recommended by [33]) are needed to understand the mechanisms linking depression and dementia, and how to differentiate between the different underlying conditions.

Another important finding of studies in this research field is the stronger effect of more severe depressive symptoms on development of dementia [9]; [10]; [14]. We only found a significant effect of MD *after* adjustment for socio-demographic characteristics, cognitive and functional status in Cox proportional hazards regression models, but neither in univariate analyses nor in binary linear regression models neglecting time to incident dementia. However, in post-hoc analyses, despite the small number of MD cases, we found a strong association of MD and incident dementia if the study population was reduced only to individuals with an MCI diagnosis at baseline which may support the hypothesis from the same underlying neuropathologic process of MCI and depression (e. g. hippocampal volume loss [34]), so that in this special case depression occurs as a prodromal stage of dementia (i.e. of Alzheimer’s disease [35]) [3]. However, related to the very small number of MD cases these results should be interpreted cautiously, and further in-depth investigation is needed.

Incidence of dementia in our study population (mean age 81.3 years) was 48 per 1,000 person years which is upper range. A systematic review showed incidence rates between 16 and 36 per 1,000 person years for the 75 to 79-year-olds and between 27 and 45 per 1,000 person years for the 80 to 84-year olds [36].

### 

#### Strengths and limitations

Before critically evaluating results, the methodical quality of the study should be emphasized: the prudent sampling strategy led to very low dropout rates, ensured representativeness of the results and minimized the likelihood of sampling errors. A comprehensive test battery including both categorical and dimensional measure of depression as well as a neuropsychological test battery and consensus conferences for diagnostics of dementia were applied, and in case of cognitive impairment, validating proxy information was gathered.

Against this, the number of MD cases was low in our study caused by a generally low prevalence of MD in old age. Furthermore, application of the CES-D is restricted in individuals with cognitive impairment [37]. To ensure validity of CES-D assessment, we decided to exclude individuals with a MMSE score below 19 (also if they had no prevalent dementia diagnosis), following a recommendation of Watson [25]. Also, the study only relies on one single measure of cognitive state at each visit by the SIDAM cognitive test battery.

Conclusively, the present study using different diagnostic measures of depression on future dementia found no clear significant association of depression and incident dementia. Further in-depth investigation with larger samples would help to understand the nature of depression in the context of incident dementia, and the mechanism linking both. Precise definitions of cases and a careful measurement using adequate sensitive and specific measures are urgently needed when investigating the relationship between disorders that influence the measurement of each other.

## References

[1] Steffens DC, Otey E, Alexopoulos GS, Butters MA, Cuthbert B, et al (2006) Perspectives on depression, mild cognitive impairment, and cognitive decline. Arch Gen Psychiatry 63: 130–138. 16461855 10.1001/archpsyc.63.2.130

[2] Jorm AF (2001) History of depression as a risk factor for dementia: an updated review. Aust N Z J Psychiatry 35: 776–781. 11990888 10.1046/j.1440-1614.2001.00967.x

[3] Panza F, Frisardi V, Capurso C, D'Introno A, Colacicco AM, et al (2010) Late-life depression, mild cognitive impairment, and dementia: possible continuum? Am J Geriatr Psychiatry 18: 98–116. 20104067 10.1097/JGP.0b013e3181b0fa13

[4] Butters MA, Young JB, Lopez O, Aizenstein HJ, Mulsant BH, et al (2008) Pathways linking late-life depression to persistent cognitive impairment and dementia. Dialogues Clin Neurosci 10: 345–357. 18979948 10.31887/DCNS.2008.10.3/mabutters PMC2872078

[5] Saczynski JS, Beiser A, Seshadri S, Auerbach S, Wolf PA, et al (2010) Depressive symptoms and risk of dementia: the Framingham Heart Study. Neurology 75: 35–41. 20603483 10.1212/WNL.0b013e3181e62138 PMC2906404

[6] Berger AK, Fratiglioni L, Forsell Y, Winblad B, Backman L (1999) The occurrence of depressive symptoms in the preclinical phase of AD: a population-based study. Neurology 53: 1998–2002. 10599771 10.1212/wnl.53.9.1998

[7] Wilson RS, Barnes LL, Mendes de Leon CF, Aggarwal NT, Schneider JS, et al (2002) Depressive symptoms, cognitive decline, and risk of AD in older persons. Neurology 59: 364–370. 12177369 10.1212/wnl.59.3.364

[8] Becker JT, Chang YF, Lopez OL, Dew MA, Sweet RA, et al (2009) Depressed mood is not a risk factor for incident dementia in a community-based cohort. Am J Geriatr Psychiatry 17: 653–663. 19634208 10.1097/jgp.0b013e3181aad1fe PMC2714703

[9] Dal Forno G, Palermo MT, Donohue JE, Karagiozis H, Zonderman AB, et al (2005) Depressive symptoms, sex, and risk for Alzheimer's disease. Ann Neurol 57: 381–387. 15732103 10.1002/ana.20405

[10] Chen R, Hu Z, Wei L, Qin X, McCracken C, et al (2008) Severity of depression and risk for subsequent dementia: cohort studies in China and the UK. Br J Psychiatry 193: 373–377. 18978315 10.1192/bjp.bp.107.044974

[11] Devanand DP, Sano M, Tang MX, Taylor S, Gurland BJ, et al (1996) Depressed mood and the incidence of Alzheimer's disease in the elderly living in the community. Arch Gen Psychiatry 53: 175–182. 8629893 10.1001/archpsyc.1996.01830020093011

[12] Geerlings MI, Schoevers RA, Beekman AT, Jonker C, Deeg DJ, et al (2000) Depression and risk of cognitive decline and Alzheimer's disease. Results of two prospective community-based studies in The Netherlands. Br J Psychiatry 176: 568–575. 10974964 10.1192/bjp.176.6.568

[13] Ganguli M, Du Y, Dodge HH, Ratcliff GG, Chang CC (2006) Depressive symptoms and cognitive decline in late life: a prospective epidemiological study. Arch Gen Psychiatry 63: 153–160. 16461857 10.1001/archpsyc.63.2.153

[14] Heser K, Tebarth F, Wiese B, Eisele M, Bickel H, et al.. (2012) Age of major depression onset, depressive symptoms, and risk for subsequent dementia: results of the German Study on Ageing, Cognition, and Dementia in Primary Care Patients (AgeCoDe). Psychol Med: In press. 10.1017/S0033291712002449 23137390

[15] Riedel-Heller SG, Busse A, Aurich C, Matschinger H, Angermeyer MC (2001) Prevalence of dementia according to DSM-III-R and ICD-10: results of the Leipzig Longitudinal Study of the Aged (LEILA75+) Part 1. Bri J Psychiatry 179: 250–254. 10.1192/bjp.179.3.250 11532803

[16] Riedel-Heller SG, Konig HH (2011) [Occurence and costs of cognitive disorders in Germany]. Psychiatr Prax 38: 317–319. 21969211 10.1055/s-0031-1276937

[17] Luppa M, Gentzsch K, Angermeyer MC, Weyerer S, Konig HH, et al (2011) [Gender-specific predictors of institutionalisation in the elderly–results of the Leipzig longitudinal study of the aged (LEILA 75+)]. Psychiatr Prax 38: 185–189. 20687014 10.1055/s-0030-1248496

[18] Brauns H, Steinmann S (1999) Educational Reform in France, West-Germany and the United Kingdom: Updating the CASMIN Educational Classification. ZUMA-Nachrichten 44: 7–45.

[19] Schneekloth U, Potthoff P, Piekara R, von Rosenbladt B (1996) [Help and need of care in institutions. Results of the representative research project 'Potentials and limitations of independent living'.]. Stuttgart: Kohlhammer.

[20] Lawton MP, Brody EM (1969) Assessment of older people: self-maintaining and instrumental activities of daily living. Gerontologist 9: 179–186. 5349366

[21] Folstein MF, Folstein SE, McHugh PR (1975) “Mini-mental state”. A practical method for grading the cognitive state of patients for the clinician. J Psychiatr Res 12: 189–198. 1202204 10.1016/0022-3956(75)90026-6

[22] Radloff LS (1977) The CES-D scale: A self-report depression scale for research in the general population. Appl Psychol Meas 1: 385–401.

[23] Hautzinger M, Bailer M, Hofmeister D, Keller F (2012) Allgemeine Depressions- skala: Manual. 2. überarbeitete und neu normierte Auflage. Göttingen: Hogrefe Verlag.

[24] Weyerer S, Geiger-Kabisch C, Denzinger R, Pfeifer-Kurda M (1992) Die deutsche Version der CES-D Skala. Ein geeignetes Instrument zur Erfassung von Depressionen bei älteren Menschen. Diagnostica 38: 354–365.

[25] Watson LC, Pignone MP (2003) Screening accuracy for late-life depression in primary care: a systematic review. J Fam Pract 52: 956–964. 14653982

[26] Spitzer RL, Williams JBWGM (1987) Structured clinical interview for DSM-III-R (SCID). New York: Biometric Research Department, NYS Psychiatric Institute.

[27] Zaudig M, Mittelhammer J, Hiller W, Pauls A, Thora C, et al (1991) SIDAM–A structured interview for the diagnosis of dementia of the Alzheimer type, multi-infarct dementia and dementias of other aetiology according to ICD-10 and DSM-III-R. Psychol Med 21: 225–236. 2047500 10.1017/s0033291700014811

[28] American Psychiatric Association (1994) Diagnostic and Statistical Manual of Mental Disorders (4th edn) (DSM-IV). Washington, DC: APA.

[29] Hughes CP, Berg L, Danziger WL, Coben LA, Martin RL (1982) A new clinical scale for the staging of dementia. Br J Psychiatry 140: 566–572. 7104545 10.1192/bjp.140.6.566

[30] Winblad B, Palmer K, Kivipelto M, Jelic V, Fratiglioni L, et al (2004) Mild cognitive impairment - beyond controversies, towards a consensus: report of the International Working Group on Mild Cognitive Impairment. Journal of Internal Medicine 256: 240–246. 15324367 10.1111/j.1365-2796.2004.01380.x

[31] Potter GG, Steffens DC (2007) Contribution of depression to cognitive impairment and dementia in older adults. Neurologist 13: 105–117. 17495754 10.1097/01.nrl.0000252947.15389.a9

[32] Ownby RL, Crocco E, Acevedo A, John V, Loewenstein D (2006) Depression and risk for Alzheimer disease: systematic review, meta-analysis, and metaregression analysis. Arch Gen Psychiatry 63: 530–538. 16651510 10.1001/archpsyc.63.5.530 PMC3530614

[33] Coyne JC, de Voogd JN (2012) Are we witnessing the decline effect in the Type D personality literature? What can be learned? J Psychosom Res 73: 401–407. 23148805 10.1016/j.jpsychores.2012.09.016

[34] Videbech P, Ravnkilde B (2004) Hippocampal volume and depression: a meta-analysis of MRI studies. Am J Psychiatry 161: 1957–1966. 15514393 10.1176/appi.ajp.161.11.1957

[35] Sweet RA, Hamilton RL, Butters MA, Mulsant BH, Pollock BG, et al (2004) Neuropathologic correlates of late-onset major depression. Neuropsychopharmacology 29: 2242–2250. 15354182 10.1038/sj.npp.1300554

[36] Fratiglioni L, De Ronchi D, Aguero-Torres H (1999) Worldwide prevalence and incidence of dementia. Drugs Aging 15: 365–375. 10600044 10.2165/00002512-199915050-00004

[37] Matschinger H, Schork A, Riedel-Heller S, Angermeyer MC (2000) On the application of the CES-D with the elderly: dimensional structure and artifacts resulting from oppositely worded items. Int J Methods Psychiatr Res 9: 199–209.

